# Supplementary material for: Modest Reduction in CAG Repeat Length Rescues Motor Deficits but Not Purkinje Cell Pathology and Gliosis in Spinocerebellar Ataxia Type 1 Mice
Source: Neuroglia. Author manuscript; Available in PMC 2026 Aug 25. (PMC13501978; doi:10.3390/neuroglia4010005)
Supplement: Supplementary file 1 [file NIHMS2200524-supplement-Supplementary_Material.pdf]

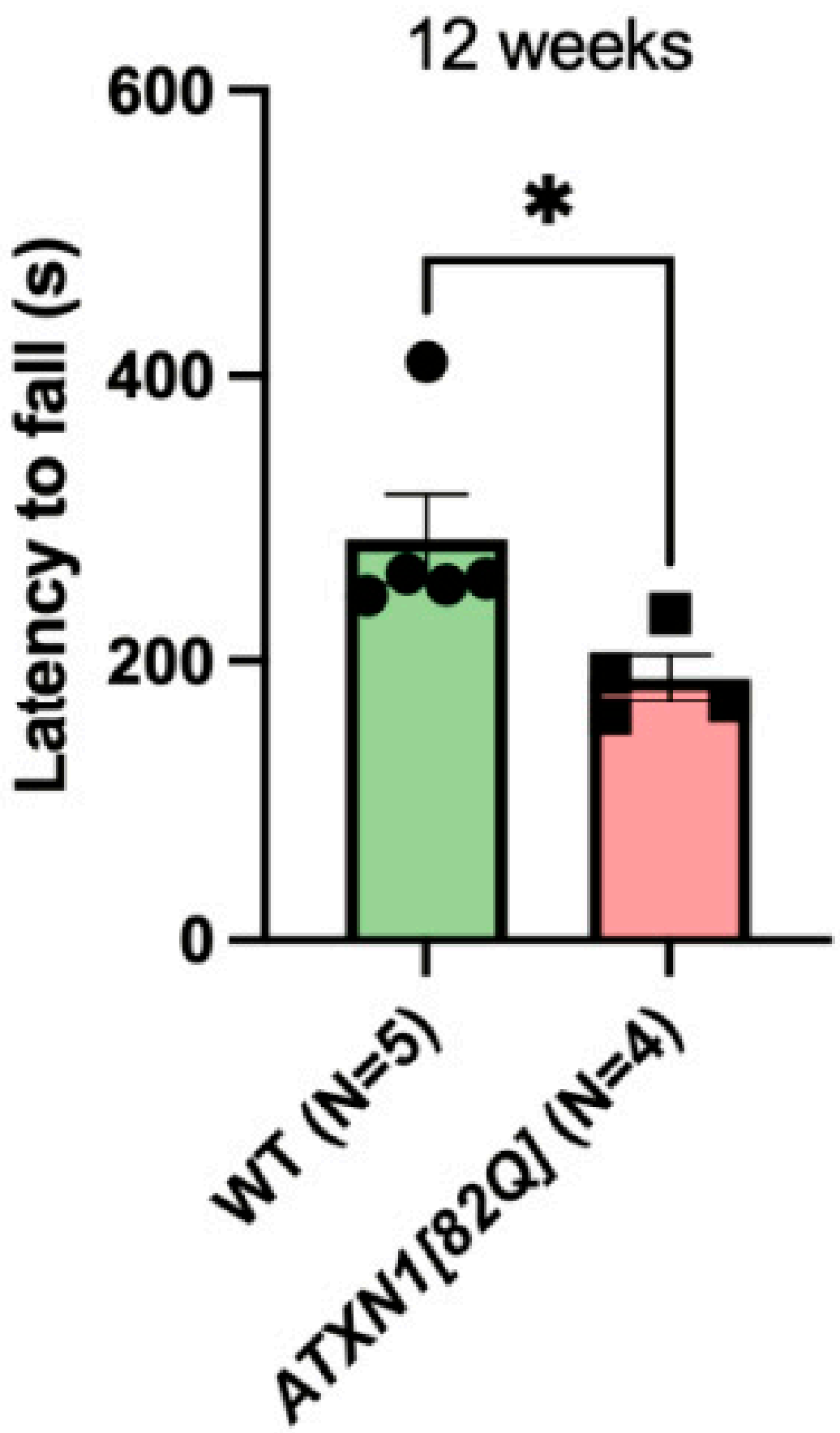

Supplementary Figure S1. Rotarod performance of ATXN1[82Q] mice is impaired at 12 weeks. Rotarod performance of mice at 12 weeks of age. Data are presented as mean ± SEM with average values for each mouse represented by a dot. * $P < 0.05$ using Student's t-test. Error bars = S.E.M.

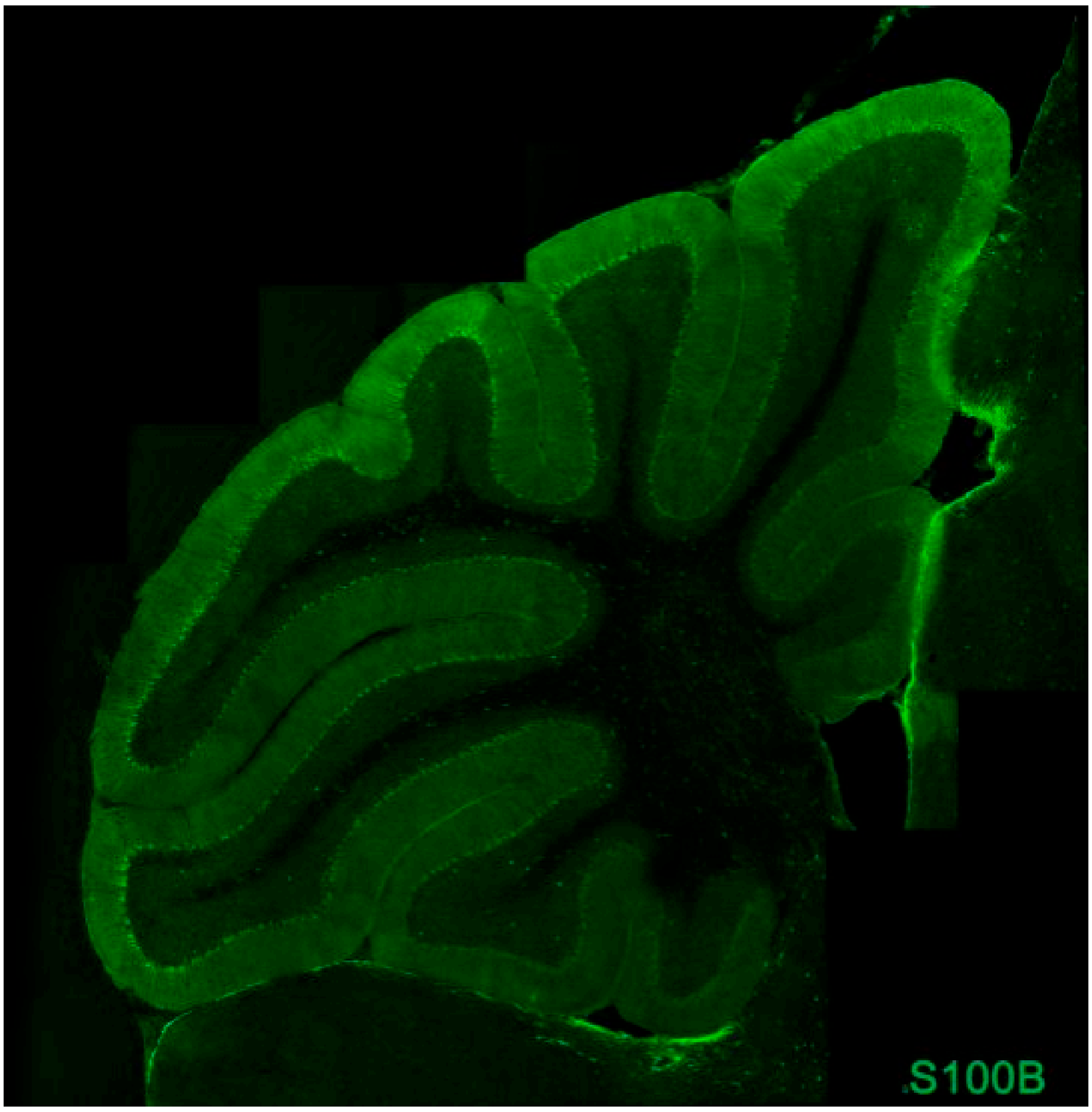

Supplementary Figure S2. Selective recombination in cerebellar astrocytes in Slc1a3-CreERT upon tamoxifen injection. Slc1a3-CreERT mice were crossed with reporter mice that have loxP sites surrounding the STOP codon in front of the TdTomato gene. TMX injection resulted in TdTomato signal in astroglial cells, marked by expression of S100B.

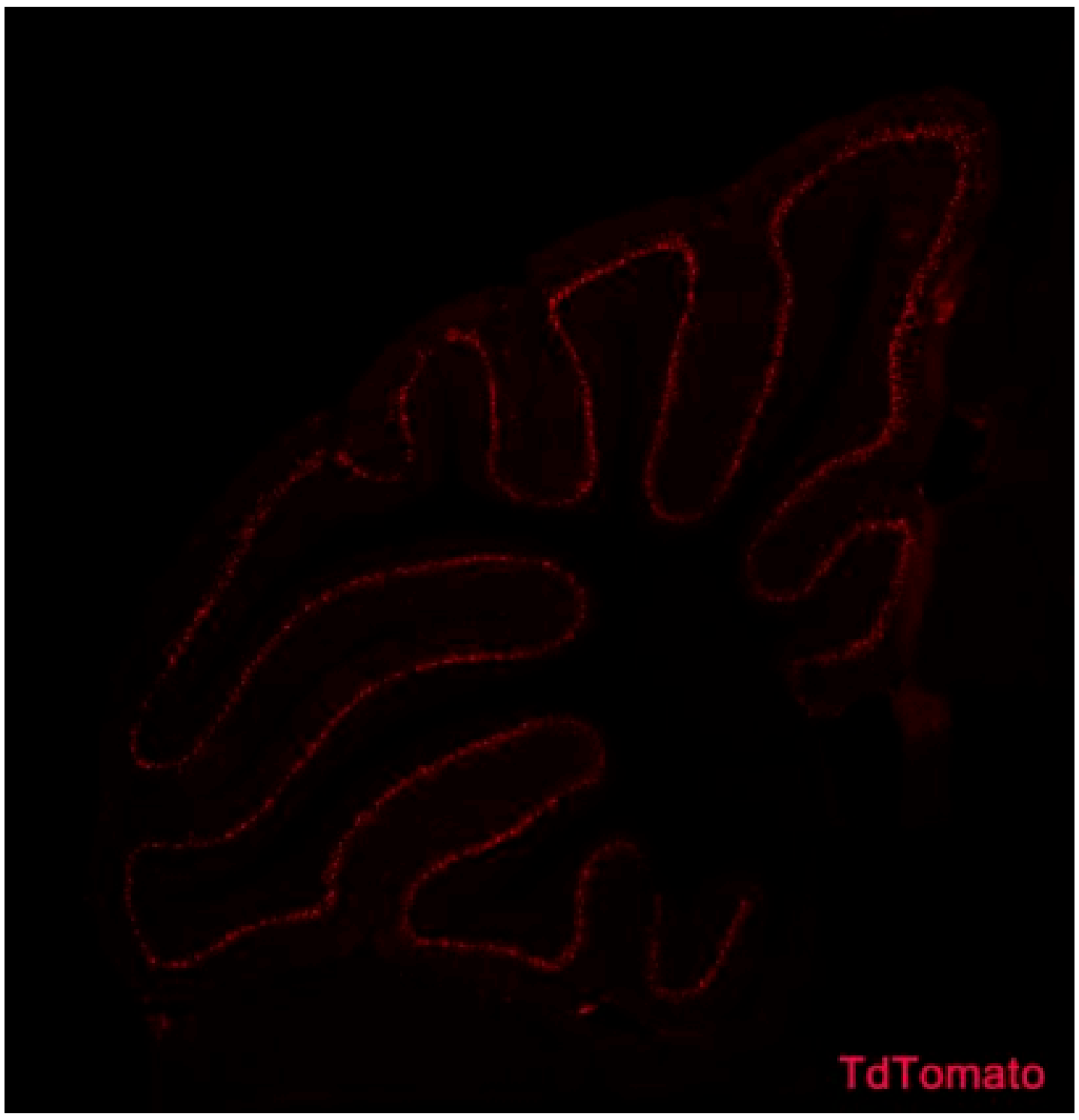

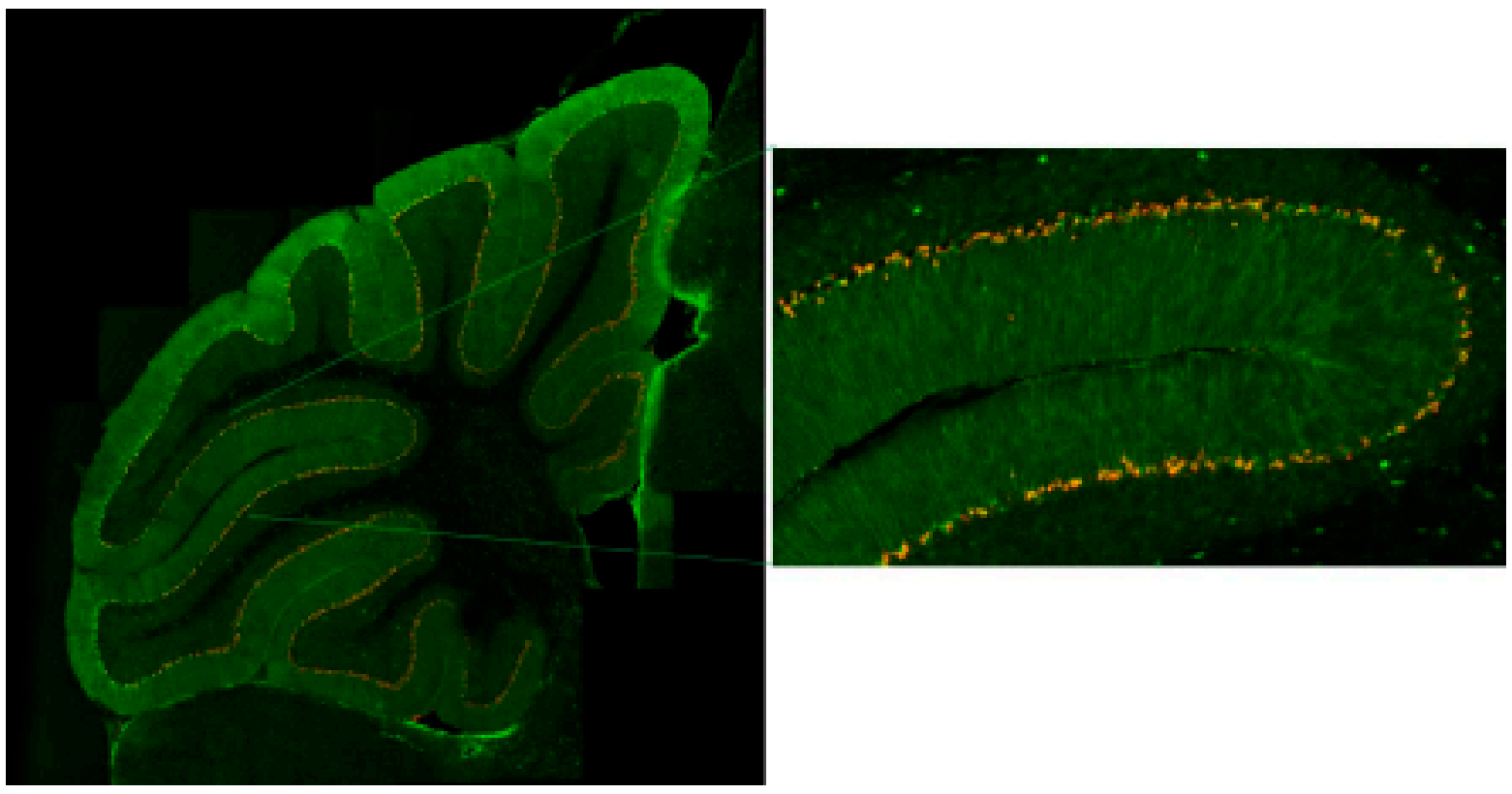

Supplementary Figure S3. Representative GFAP immunofluorescence staining in the cerebellar molecular layer of 22-26-weeks-old mice.

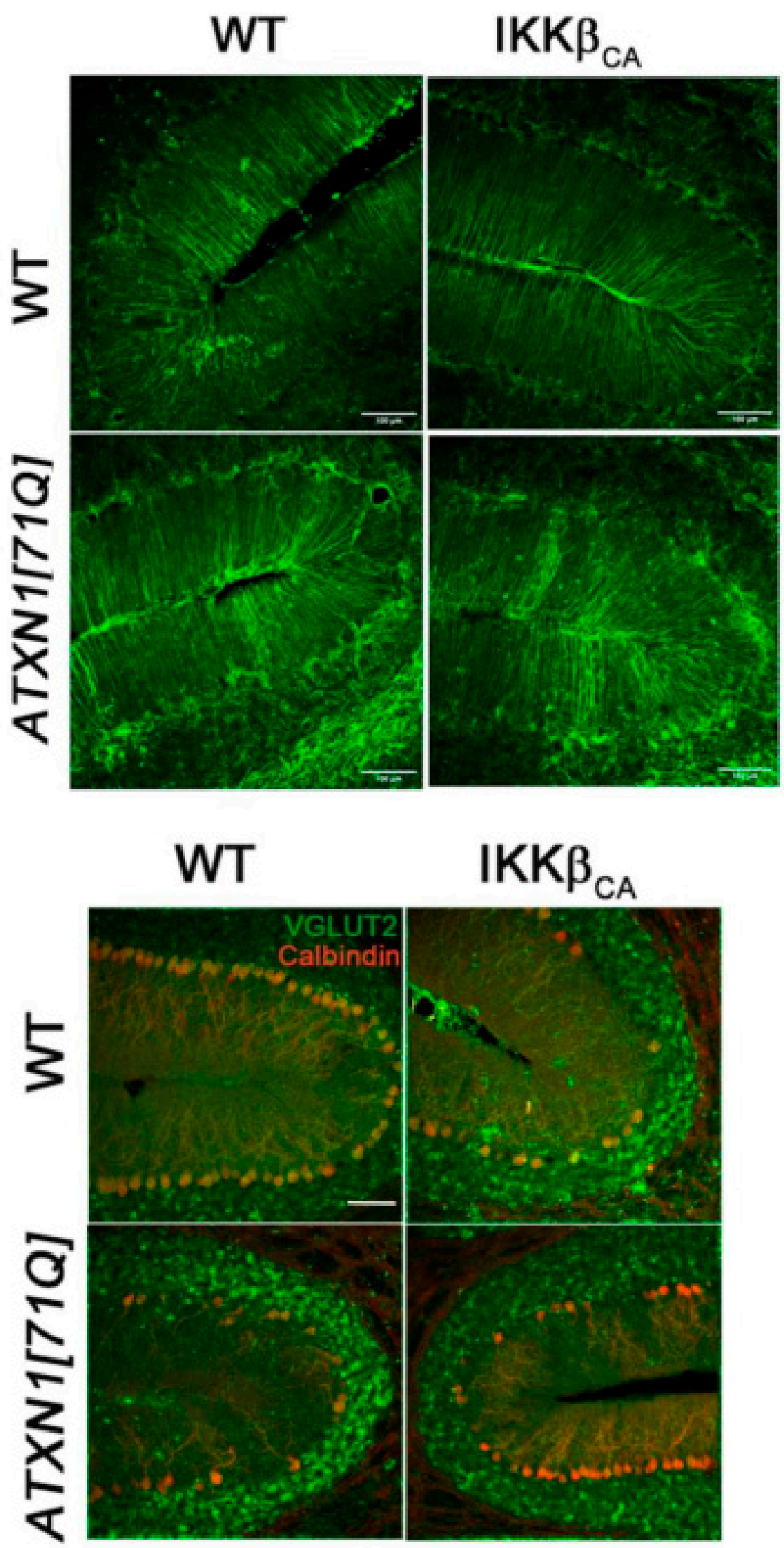

Supplementary Figure S4. Representative VGLUT2 (green) and calbindin(red) immunofluorescence staining in the cerebellar molecular layer of 12-weeks-old mice.

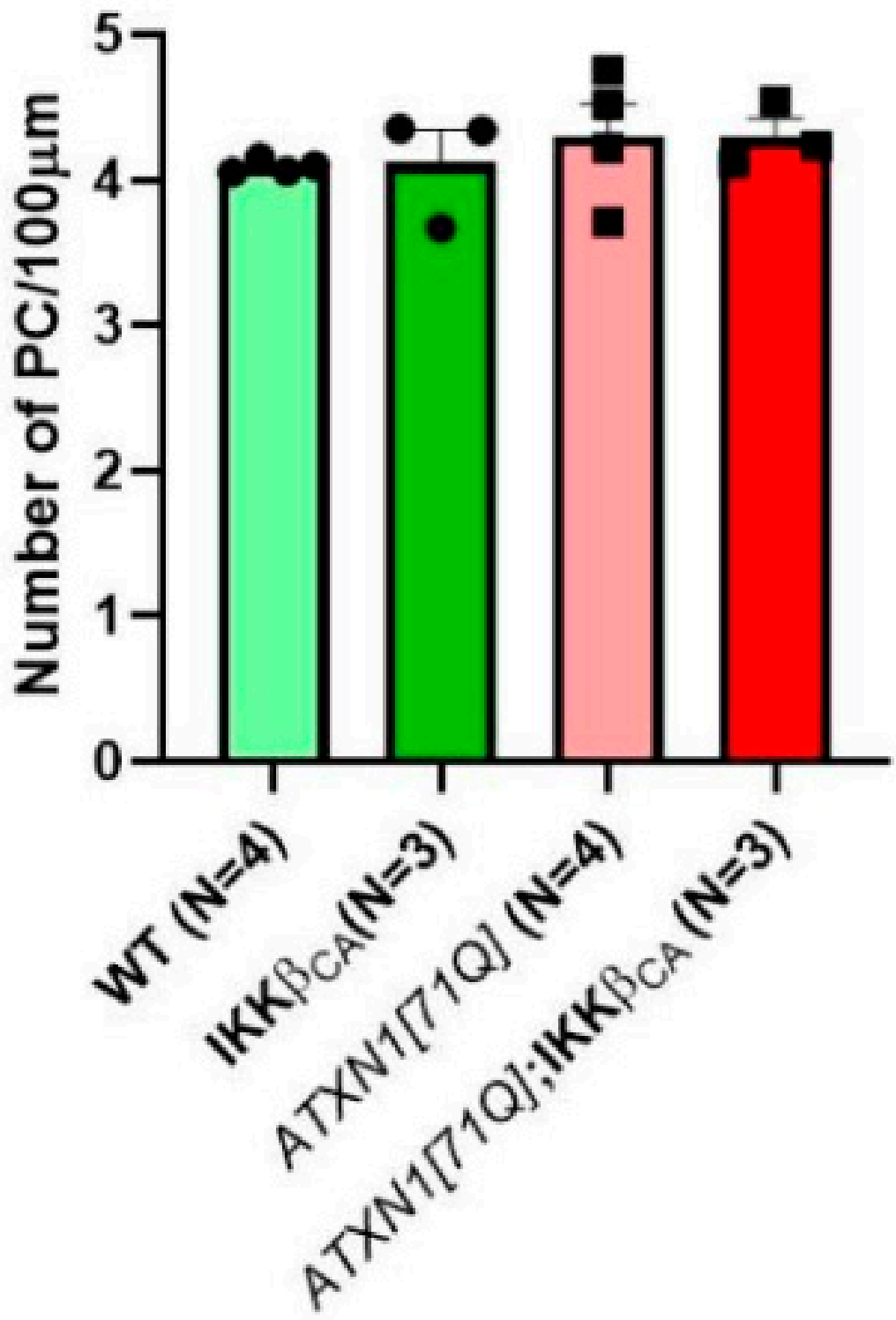

Supplementary Figure S5. Purkinje cell number is not altered in ATXN1[71Q] mice. Image J was used to quantify the number of calbindin positive Purkinje cells at 22-26 weeks. Data are presented as mean ± SEM with average values for each mouse represented by a dot. No statistical differences using one-way ANOVA with Bonferroni's multiple comparison test. Error bars = S.E.M.
